# Variations in perceptions of parenting role related to children’s physical activity and sedentary behaviours – a qualitative study in a Northern European context

**DOI:** 10.1186/s12889-021-11537-7

**Published:** 2021-08-14

**Authors:** Susanne Andermo, Helena Rydberg, Åsa Norman

**Affiliations:** 1 Department of Global Public Health, Karolinska Institutet, SE-171 77 Stockholm, Sweden; 2 Department of Neurobiology, Care Sciences and Society, Karolinska Institutet, Stockholm, Sweden; 3 Department of Clinical Neuroscience, Karolinska Institutet, SE-171 77 Stockholm, Sweden

**Keywords:** Children, Parental support, Physical activity, Exercise and sedentary

## Abstract

**Background:**

The aim of the study was to increase understanding of the variation in parental perceptions of their roles and responsibilities in relation to children’s physical activity and sedentary behaviours.

**Methods:**

This qualitative study was based on data from the Healthy School Start intervention study II, in the form of recorded motivational interviewing (MI) sessions with mothers and fathers participating in the intervention. Forty-one MI sessions where parents discussed physical activity and/or sedentary behaviour were selected for analysis. Data analysis was performed using a phenomenographic approach.

**Results:**

Three categories describing a structural relationship of parents’ different views on their own role in relation to their child’s habits were identified: 1) The parent decides – Child physical activity according to my beliefs and views as a parent and where I, as a parent, decide, 2) Parent-child interaction – child physical activity is formed in interaction between me as a parent and my child or 3) The child/someone else decides – The child or someone other than me as a parent decides or has the responsibility for my child’s physical activity. All three categories included four subcategories of specific activities: organised activity, activity in everyday life, being active together and screen time, describing practical approaches used in each of the three categories.

**Conclusions:**

This study found variation in mothers’ and fathers’ perceptions of their roles and responsibilities for their child’s physical activity and sedentary behaviours related to specific types of activities. The results indicate areas where parents need support in how to guide their children and how parental responsibility can have a positive influence on children’s physical activity and sedentary habits.

## Background

Regular physical activity has been associated with a range of physical, social and mental health benefits for both adults and children [1–4]. For children, physical activity is essential for the promotion of healthy musculoskeletal, cardiovascular, and neuromuscular development, and for healthy weight management [5], as well as increased psychosocial health; such as self-concept [3, 6] and self-esteem [7]. The World Health Organisation (WHO) recommends an average of 60 min/day of moderate-to-vigorous intensity aerobic physical activity across the week for children aged 5 to 17 years old [8]. However, few children reach recommended levels of physical activity [9, 10], and physical activity declines with age [9]. There are also socioeconomic and gender inequalities seen with regards to physical activity [9, 11]. International data has shown that children of parents with a high level of education participate more in organised sport than children of parents with low education [12, 13], although studies from Sweden have shown inconclusive results [13, 14]. Internationally, girls with an immigrant background are identified as least physically active and participate least in organised sport [10, 11]. In addition, sex differences have been found, where boys are more active than girls [15]. Oher correlates of physical activity in children include physical activity preferences, intention to be active, time spent outdoors, previous physical activity and access to facilities [16]. Considering that early physical activity and sedentary behaviours tend to track into adulthood [17], there is an urgent need to further the understanding on how to promote and establish physical activity habits at an early age.

Children’s physical activity levels are influenced by individual, social, cultural and environmental factors [18–20], and parents play an essential role in influencing physical activity behaviours in younger children [21]. Several factors are of importance regarding parenting in relation to children’s healthy behaviours, such as parenting styles, practices, attitudes, and perceptions [22–27]. Regarding parenting styles, four different parenting styles have been identified across the two dimensions demandingness and responsiveness, where demandingness signals parental control, and responsiveness signals affective warmth. The four styles are: 1) the authoritative style where parental expressions are high on both dimensions, 2) the authoritarian style where parental expressions are high on demandingness and low on responsiveness, 3) the indulgent/permissive style where parental expressions are low on demandingness and high on responsiveness, and 4) the uninvolved/neglectful style where parental expressions are low on both dimensions [28]. Parenting practices are described as specific behaviours performed by the parent in relation to the child in specific situations. Specific parenting practices related to children’s physical activity have been described in the literature e.g. praising, monitoring, or encouraging child physical activity, pressuring the child to engage in physical activity, or modelling physical activity [29, 30]. Together, parenting practices entail *what* the parents do, while parenting styles are *how* they do it – the parenting style being the context within which parenting practices are carried out [30].

Research regarding parenting styles has identified the authoritative parenting style as being associated with the most positive health outcomes in children [18, 22, 23, 31, 32]. The authoritative parenting style has also been associated with greater physical activity in children [22, 23], whereas the neglectful style has been associated with lower child physical activity [33]. Regarding sedentary screen time, the authoritative parenting style has been associated with lower sedentary screen time [32, 34], whereas the neglectful, permissive and authoritarian parenting styles all have been associated with higher screen time in different studies/populations [32, 35]. Studies focusing on parenting practices in relation to children’s physical activity have found the most consistent evidence for positive associations between the specific parenting practices of modelling physical activity behaviour, and parental logistic support for physical activity and children’s physical activity [24, 25]. Additional, but less conclusive, evidence has been found regarding associations between parental encouragement and children’s physical activity [25]. Regarding relationships between parenting practices and children’s screen time, there is support for an association between parenting restrictive practices, i.e. parents setting limits or rules for the amount of screen time allowed, and less screen time in children [25, 36]. Furthermore, parents reversed modelling of screen time behaviour, i.e. parents practicing a lot of screen time themselves, has been associated with more child screen time [25, 37].

Research regarding parental attitudes towards physical activity includes several aspects, such as perceived importance, enjoyment, and perceived availability. Here, research has shown that parents’ beliefs that physical activity is important and has been positively associated with children’s physical activity [26]. Also, parental enjoyment of physical activity and perceived availability of sporting clubs [38] and financial support [39] have been positively associated with children’s physical activity. Thomson et al. [27] conducted interviews with parents in the UK and showed that although parents valued family engagement during physical activity, especially as a means for improving parent-child communication, they also reported a variety of barriers, such as a busy lifestyle, diverse ages and interests of children and adults, bad weather, and lack of access to facilities.

Considering that parents are so important for children’s healthy behaviours, the parent’s sense of responsibility regarding their own role in their child’s physical activity behaviours is of essential importance to study. However, this aspect of parenting in relation to children’s activity behaviours has received little prior attention. In one study, parents of 11- to 12-year-old children in New Zealand had difficulties defining what parental responsibility for children’s physical activity encompassed. Instead, they described behaviours that reflected responsibility, such as logistic support, role modelling, and prioritisation of children’s needs. Parents’ expression of responsibility varied in relation to their own time and financial limits, e.g. parents who lacked time were reluctant when it came to child physical activity that demanded their parental support, but also in relation to child-parent relations, e.g. where child commitment to an activity would require more parental responsibility to make sure that the activity happened [40]. In addition, a study of parents in the UK found that some parents viewed the school as having the primary responsibility for their child’s physical activity [41]. As the concept of parental responsibility for children’s physical activity is only vaguely described in the literature, in this study we use parental responsibility for the child’s physical activity to mean the parent’s view of their own position in relation to ensuring that the child’s physical activity takes place. Additionally, the parental role refers to the actual part the parent views themself playing in their child’s physical activity behaviour, e.g. an active or passive role.

Although parenting in relation to children’s physical activity and sedentary behaviours has gained increased attention during the latter years, as presented above, it has been questioned whether current concepts and measurements related to parenting capture all important aspects of parenting related to children’s physical activity behaviours [42]. Commonly used methods to measure parenting include methods such as questionnaires and interviews. The use of unobtrusive methods, collected without or with minimal interference with the participant, for example archival records [43], have the possibility to shed light on previously unidentified aspects of parenting of importance to the field. Also, the use of a method to analyse data which allows for the identification of a range of different aspects of parenting may also further inform the field. Phenomenography is a qualitative methodology which seeks to explore variation in experiences of a certain phenomenon among a specific group [44], e.g. experiences of parenting in relation to children’s physical activity among a particular group of parents. In addition, though parenting in relation to physical activity has received some attention, parenting in relation to sedentary behaviours e.g. restriction of sedentary behaviours such as screen time has been studied less [21]. Also, the majority of studies on parenting in relation to children’s physical activity and sedentary behaviours have been conducted primarily with mothers. Studies including fathers are called for [45]. Furthermore, differences in parenting related to culture have been suggested [30], and a pattern of styles has been suggested for the Northern European context specifically [18]. The four-season climate, including cold winters, may also influence parenting in relation to children’s physical activity in the Northern European context. In addition, there is evidence to suggest that there are socioeconomic variations in parenting where families with a lower socioeconomic position may be in need of more support to engage in positive parenting for children’s healthy behaviours [46]. While the need for further understanding of cultural and socioeconomic functions and variability in parenting has been voiced [46], few studies on parenting related to children’s physical activity and sedentary behaviours have been conducted in a Northern European setting.

In summary, parents play an important role for their child’s physical activity behaviours, but there is a lack of studies exploring parents’ sense of responsibility for their physical activity and sedentary behaviours in general and in a Northern European setting specifically. In addition, very few studies on parenting related to children’s physical activity have included fathers, and few studies have used methods that shed light on the variation in important aspects of parents’ sense of responsibility. By identifying parents’ variation of experiences regarding their children’s physical activity and sedentary behaviours, we can better understand the type of interventions and support that these groups need.

The aim of the study was therefore to explore variations in parental perceptions of their roles and responsibilities in relation to children’s physical activity and sedentary behaviours. This study applied a phenomenographic approach to explore variations in parental perceptions using data from real-life behaviour modification sessions with parents in connection with a child’s health visit in school in disadvantaged areas in Sweden.

## Methods

### Setting

This study uses data from the Healthy School Start (HSS) trial. The HSS is a child health promotion and obesity prevention intervention for children starting school which focuses on parental support [47]. The intervention was conducted as a cluster randomized trial in 2012–2013 in 31 pre-school classes (children aged 5 to 7), that were randomised to intervention- or control groups, in three areas within Stockholm county, Sweden. The areas were considered to be disadvantaged based on employment and education levels, and were eligible for government initiatives to support socioeconomic development [48]. The intervention was carried out during 6 months and encompassed three components. 1. Health information to parents regarding children’s diet, physical activity and sleep. 2. One or two (depending on the wishes of the parents) motivational interview (MI) sessions with a professional MI counsellor where parents had the opportunity to focus on a change regarding their child’s diet or physical activity that they wanted to implement in the home environment. The second of the two MI sessions was a follow-up session offered 3 months after the first one. 3. Ten classroom lessons performed by the teachers. Each of the ten lessons was accompanied by a brief activity-based home assignment for the family to complete together [49].

### Design

Data used in the study comprised of recorded and transcribed sessions of MI from the HSS intervention study; the present research questions were developed a posteriori. A qualitative phenomenographic approach [44] was used. The phenomenographic inductive approach was considered suitable as it is used to explore variations in experiences of one or several phenomena, to which each participant within the target group may contribute several ways of experiencing [44]. In this study, the phenomenon in focus pertained to parents’ perceptions of their roles and responsibilities in relation to their child’s physical activity and sedentary behaviours. Phenomenography is based on a non-dualistic ontology, which means that the intrinsic experience and the outer world are constantly influencing each other. Phenomenography focuses specifically on the core of the relationship between the inner and the outer world. This core is called the second-order perspective, which is expressed as an implicit and underlying experience that is taken for granted by the participant. The results of a phenomenographic study, and thus the variations in experience, are described according to categories comprising qualitatively different ways of experiencing a phenomenon. The categories are then related to each other in a logical structure, which in turn describes an underlying common pattern of the categories [44]. This structure is crucial to phenomenography, as each individual interview cannot be understood on its own, but must be seen in the context of the other units of data [44].

In this study, we applied the phenomenographic approach to MI sessions with parents collected within the HSS. The parents were aware that the sessions were audio-recorded, and that the sessions were part of the HSS research project. However, they were not aware of the specific research question of this study as the present research question, as mentioned, was developed later. The MI session can, therefore, be considered to have been collected in a semi-unobtrusive manner. Unobtrusive data collection, such as audio recordings or archival records, does not influence the participant, in contrast to data collection methods where participants are asked questions directly related to a research question, such as in questionnaires or interviews [50]. This study used audio-recorded and transcribed MI sessions where the parent reflected on their child’s physical activity and/or screen behaviours, which can be considered to represent real-life situations of parents’ reflections about their children’s behaviours.

### Selection of participants

In this study, parents in the intervention group of the HSS intervention comprised the sample of participants. Inclusion criteria for this specific study were: attending the first MI session, and a focus on physical activity or sedentary behaviour in this first MI session. In the HSS, a total of 146 first sessions of MI were conducted, of which 41 focused on child physical activity or screen behaviour. All 41 MI sessions were included in this study. The MI sessions lasted between 12 and 61 min with a mean of 27 min. A roughly equal number of sessions were conducted with mothers only (*n* = 21) as with fathers only (*n* = 17), and three sessions were conducted with couples (mother and father together) (Table 1). Most families lived in a rented flat (*n* = 26), were born outside the Nordic region (*n* = 30) and had studied at university level (*n* = 29) although they did not necessarily have a university degree. All families resided in areas that had been identified as disadvantaged by the Swedish government (based on employment and education levels) and that were targeted by government initiatives to support socioeconomic development [48]. Socioeconomic position can be measured using a number of indicators on individual, family, or area level such as individual or family level of education, income, occupation, housing tenure status, or area level of deprivation [51]. Education at an individual level constitutes a commonly used indicator but using education as a sole indicator of socioeconomic position in complex situations, such as when migration is involved, may be misleading as the level of affluence associated with a given level of education in the previous home country may not correspond to the level of affluence in the new host country, especially if employment opportunities are worsened [51]. In light of the situation of the families in the study in terms of migration status, education, housing, region of birth, and area of residence, we consider the families to lead lives in a setting of lower, but complex, socioeconomic position, of significant interest to the research field.

**Table 1:** Characteristics of participating parents and their children

**Parents *n *= 41**	
Mothers/fathers/couples (*n*)	21/17/3
Highest education (*n*):	
≤ 9 years (Primary school)	6
10–12 years (	3
> 12 years (University)	29
Unknown	3
Housing conditions (*n*)	
Rental flat	26
Owned flat	7
Owned house	5
Unknown	3
Region of birth (*n*)	
Nordic region^a^	8
Europe	3
Asia	17
Africa	10
Unknown	3
**Children**	
Boys/girls (*n*)	21/20
School classes represented	14

^a^ Including Sweden, Norway, Denmark, Finland and Iceland

### Data collection

During the HSS trial, parents attended MI sessions that were conducted as an extension of compulsory health visits with the school nurse. All children in Sweden visit the school nurse during their first year of school, and parents are expected to accompany their child to the visit. During the health visit, the child’s weight development is monitored and habits are discussed. Parents attended the MI sessions as part of the health visit without any prior expectation of the sessions or specific interest in talking about their children’s physical activity behaviours. Only data from the first, out of two possible MI sessions, are included in this study. The first sessions were deemed more suitable to address the aim of this study, as these sessions were longer and contained the most data. In the first session, parents discussed more at length about the target behaviour (i.e. a desired behavioural change or sustaining a healthy physical activity behaviour), whereas the second MI session served as a follow-up. Moreover, not all parents participated in the second session. Examples of target behaviours that the parents raised in the included MI sessions were: increasing physical activity in everyday life, engaging in physical activity together as a family, engaging the child in organised physical activity (e.g. sports club), introducing or adhering to screen time rules, or motivating activity indoors. The MI sessions were conducted by two trained MI counsellors who demonstrated acceptable fidelity to motivational interviewing, i.e. competence in performing motivation interviewing, measured objectively [47]. All sessions were conducted in Swedish.

MI is a client-centred style of communication with the aim of supporting a person’s specific behaviour change [52]. Being a person-centred method, the sessions where MI is used are characterised by being flexible, as the counsellor focuses the session content according to the needs of the specific person receiving the session. During the HSS intervention, parents participating in the MI sessions had the opportunity to reflect on their child’s physical activity and/or screen behaviour and potential ways to change that behaviour. The parents were supported by the counsellor in a personalised manner, according to the specific family situation and the perceived need by the parent, where the parent’s own thoughts and feelings in relation to the target behaviour were evoked and explored. Evocation of a person’s thoughts and feelings is intrinsic to MI as a method. The MI counsellor uses open-ended questions and reflections, i.e. mirrors what the person has said or meant, in the evocation process to support the person’s own intrinsic motivation for behaviour modification, and come up with his or her own steps for how to realise the modification. As the parent typically started the session by describing the child’s overall behaviour and then moved on to the child behaviour the parent wished to focus the session on,, the sessions are rich in data regarding parental reflections on their own parenting and on the child’s behaviour. This makes the sessions useful for studying parents’ perceptions on their roles and responsibilities related to their child’s physical activity behaviours.

### Data analysis

HR, SA, and ÅN conducted the analysis, and all sessions were transcribed by one of the three authors. Transcriptions were made verbatim and intonation was marked in italics. The analysis was conducted in accordance with the steps suggested by Åkerlind [53, 54]. Transcripts were initially treated as a whole and later in the analysis as larger chunks of text in order to keep the context of important statements, as suggested by Åkerlind [53]. Firstly, the authors got acquainted with the data by reading the whole transcripts. In this initial reading, the authors gained a first view of similarities and differences between and within each transcript. Secondly, authors marked chunks of text of relevance for the study aim and made notes on the content of the text. In this step, the authors began to search for patterns related to the study aim through the phenomenographic second order perspective, i.e., underlying perceptions about parental roles and responsibilities in relation to children’s physical activity and sedentary behaviours that were taken for granted by the parent. Thirdly, the authors made summaries of each transcript, where key topics of importance for the study aim were emphasised (e.g. parental sense of responsibility). In the fourth step, the authors read and grouped the summaries according to similarities and differences in these key topics (e.g. if parental sense of responsibility related to different activities seemed to be more or less pronounced). As several transcripts contained two or more parental strategies and thus related to more than one group, the authors then undertook an iterative process where they alternated between the whole transcripts and the summaries, until a final set of groups and the structural relationships between the groups was reached. All three authors independently analysed the data, discussed the analysis and agreed upon the final categories and sub-categories. The analysis was undertaken in Swedish and an English translation was conducted when the final set of groups and relationship was reached. In the quotes, square brackets are used to present modifications and explanations.

### Ethical considerations

Ethical approval for this study was obtained from the Regional Ethical Review Board in Stockholm, Sweden (2012/877–31/5). Informed consent was collected from all parents in writing.

## Results

Three categories describing parents’ perceptions of their roles and responsibilities related to their child’s physical activity behaviours were found in the analysis. The three categories related to each other within one structural relationship describing parents’ different views on their own role in relation to their child’s habits. The three categories were 1. The parent decides – Child physical activity according to my beliefs and views as a parent and where I, as a parent, decide, 2. Parent-child interaction – child physical activity is formed in interaction between me as a parent and my child or 3. The child/someone else decides – The child or someone other than me as a parent decides or has the responsibility for my child’s physical activity. Four subcategories of specific activities as practical strategies were repeated in each of the three categories. The subcategories related to specific kinds of activities: organised activity such as the child’s participation in a sports club, activity in everyday life such as active transport or play, activity that was conducted jointly by the parent and child, and finally sedentary behaviour in the form of screen time. Results are described in Table 2.

**Table 2:** Description of categories, and sub-categories within the structural relationship

Structural relationship	Parents’ view on the role as a parent in relation to the child’s physical activity habits		
Categories	The parent decides	Parent-child interaction	The child/someone else decides
Subcategories	• Organised activity	• Organised activity	• Organised activity
	• Everyday life	• Everyday life	• Everyday life
	• Activity together	• Activity together	• Activity together
	• Screen	• Screen	• Screen

### The parent decides – child physical activity according to my beliefs and views as a parent and where I, as a parent, decide

In this category, parents held the view that they were the ones to decide whether an activity was suitable or not according to their own preferences or perception regarding resources or their child, and the child’s view was not included.

#### Organised activity based on what the parents find suitable

Parents had a very positive view of organised activity and believed that it was the best way for their child to be active, but they had clear ideas on what made an activity suitable or not. They viewed organised activity positively for several reasons: it was seen as a solution to a decreased activity level during the winter, a way of creating an activity habit through the structure of having e.g. weekly sessions, and as it results in positive health outcomes;

*“I have been thinking of signing her up for gymnastics or something, like athletics [… ] so that there is [activity] time in winter, that she participates every time [… and] that she gets activity, movement [for] the body. And she will definitely feel good from it [… ] and it is also an advantage in her physical and mental development. (Father 1)*

In order to suit parents, the activity had to be close to home, and suit the parents’ schedule and their personal resources as a role model.

Parents also had a clear view of what the parents believed suited the child as a person, but without involving the child in these reflections. Activities could be rejected based on their view of the child’s ability to be part of a group, maturity, skills, or body composition. The parent might not register the child for an activity that the child had expressed a preference for if the parent disliked the activity, e.g. viewed ice-hockey as too rough, or considered ballet only suitable for slim children.

*She has always wanted to do ballet, but I have heard from many parents that I shouldn’t let her because it can lead to eating disorders [..] so that’s why I haven’t [signed her up]. Because she is not a slim child. Well, she’s not overweight [but] I’m afraid that she will say ‘God everyone is so slim, and I feel I am … [not]’ (Mother 1)*

#### Activity in everyday life is good thing, but as I have to arrange it, it is a challenge

Parents recognised that incorporating more activity in daily routines would be good for health reasons but identified several barriers both in the outdoor and indoor environment. Parents perceived children as less active nowadays in general and felt that it was up to them as parents to make everyday activity happen, which they found difficult. Barriers to outdoor activity included not being a good role model as a parent, e.g. due to physical inabilities, lack of energy or own preferences for being inactive. They also included time constraints, a perceived difficulty of getting out when living in an apartment building, and that it was dangerous to let children go outside in the winter cold and darkness. One parent addressed the difficulty of being home alone with several children making it hard to go out;

*When you live in an apartment there aren’t the same movement patterns as when you live in detached house or townhouse where you dare to let them go out. But as for activity, there is too little of it. Partly due to the work situation. You could say that we are almost never at home together, so there is a lot for one person to take care of [if going out with two children]. (Father 2)*

Barriers to indoor activity included the same role model and time-related barriers as above, but also a wish to not disturb neighbours and having a hard time identifying suitable indoor activities in the home.

#### Joint activity according to my preferences as a parent and what I think the child needs

Parents identified engaging in an activity together, parent and child, as a facilitator for being active, but the choice of activity was decided according to the parents’ preferences. The choice of joint activity could also be based on something the child needed to learn, like ice-skating or swimming;

*The indoor swimming pool is ok [to go to together I think], but sometimes there’s a lot of people and that’s a bit scary. I get a bit like [uncomfortable], so I have been there once with him. (Mother 2)*

#### Screen time is up to me as a parent to limit, but I feel lost

Parents had a clear idea that they were the ones to enforce limits about screen time but felt inhibited by several factors and were unsure e.g. about how much screen time was ok. Inhibiting factors included finding it difficult to fill the time with activities on weekends, or being outdoors during winter where a screen fills the time. Another barrier was the perception of the other parent’s behaviour, where the other parent might put the child in front of the TV when preparing dinner, or use a screen a lot themselves. A mother described:

*On weekends they get up at 8 a.m. and I am too tired to get up then so I tell them to turn on the TV. [… ] Two parents working full time, then you just don’t have the energy. Then I have my husband against me because he can say, after weekend breakfast, “can’t we just watch a film”. (Mother 3)*

Parents were concerned about the health consequences of screen time, e.g. effects on eyesight, becoming overweight, but they also acknowledged the educational and relaxing aspects of devices with screens.

### Parent-child interaction – child physical activity is formed in interaction between me as a parent and my child

In this category, the parents viewed their child’s physical activity as something that happened as a result of interaction between the parent and the child.

#### Organised activity based on my child’s personality and interests

Parents tried to arrange for the child to participate in organised activity based on who the child is as a person, and the child’s preferences, with the view that the child chooses him- or herself and that the activity should be fun and supportive for the child. The parents could also offer the child a range of activity options to see what fits the child, perhaps trying out several activities. Some may not be suitable due to the child being e.g. shy, and then the parent did not insist on participation. One mother said;

*I feel a bit that I want to know what she is interested in. Now I offer different things, and then she gets to see what she thinks. (Mother 4)*

#### Activity in everyday life that I want to make possible for my child but based on my child’s preferences

Parents wanted to enhance possibilities for the child to be active both indoors and outdoors and emphasised the importance of listening to the child’s wishes, providing their child with time to be active and have fun. The parents came up with innovative solutions and alternatives for indoor activity such as throwing soft balls, active games, and allowed children to play actively indoors by running or jumping on the bed;

*Find strategies for them to come up with games [indoors]. For example, they compete and chase each other, the kids. Buy toys that make them active. Jumping on the bed [… ] and we bought a basketball to put on the door. (Father 3)*

Parents also reflected on strategies to facilitate activity outdoors such as allowing for adventures on the way home from school, staying outside for some additional play after school, and allowing the child to play outdoors with friends.

#### Joint activity is good as the child needs to see the parent be active as well

Parents viewed it as important that their child saw them being active and viewed joint activity as an easy way to accomplish that. A mother reflected about the importance of joint activity;

*But I wish that we spent time outdoors more sometimes. Then I guess it’s because I don’t have the energy myself [… ] But then I think it’s good if the entire family does something. And that children see that parents do things, I think that influences a lot, that you are a role model. Because when I go to the gym or go running, they don’t see that I do it. They see when I go and when I come home, but that’s not the same. So, I think it is good to do [activities with] the whole family. (Mother 5)*

Parents recognised that joint activities should be experienced as fun by the children and could be different depending on the season. Some viewed the summer as the best for activities, but others also identified possibilities in winter such as skating or using the toboggan. Others overcame the issue of the cold in winter by engaging in joint activities indoors such as soccer or going to the swimming pool. Barriers for joint activity identified by parents included: their own energy level, having many children to look after, economic restraints, or lack of cooperation between the parents, where one parent could feel like they were left with the responsibility to be active with the children on weekends.

#### Agree upon screen time limits together with the child

Parents reflected upon their strategies for how to agree upon screen time limits together with their child. They wished to limit their child’s screen time, and described how they as parents interacted with their children in setting limits. The parents intended to limit their own use of screens, as they felt that they could not expect more from the child if they didn’t themselves act as good role models. Parents described preset rules which were met with debate from the children when first implemented, but were then quickly accepted and agreed upon. Parents also described limits set in the spur of the moment, as a result of the child not behaving as expected, when a child had used screens a lot during 1 day, or already had other fun activities during the day, making screen time unnecessary. These limits were met with different reactions from the children. In some cases they were immediately accepted, in other cases they were protested against (such as by claiming that they – the child – had nothing else to do), or in some cases they were ignored by the children. Parents described how they insisted on the limits and their intention to agree upon them together with their child, though they described difficulties on occasion, e.g. when stressed, when busy with chores or when a baby calls for attention;

*The kids are not home alone, so either I, the dad, or another adult know what they do on the screens [… ] We don’t give in easily, but now we have a little baby so it’s not always easy. Ideally, he would sit there for half an hour and then get up and then half an hour again [of screen time], that he would understand that, but he wants to see the next and the next program … (Mother 6)*

### The child/someone else decides – the child or someone other than me as a parent decides or has the responsibility for my child’s physical activity

In this category parents were of the view that their child’s physical activity is the responsibility of the child, or that the school should cater to the child’s needs for physical activity.

#### Organised activity as a possible solution for not having to activate the child myself as a parent, but also rather unnecessary as the school provides enough activity

Parents held the view that organised activity was a good solution for activating the child and put the responsibility for the child’s activity on the organiser instead of on themselves. At the same time, they thought that children have enough organised activity managed by the school, e.g. physical education and skating;

*They do [activities] all day at school, but we [parents] don’t know how to ski. They have ice-hockey, or what’s it called, skating, one a week there [at school]. She says all the time that it’s fun. (Father 4)*

Also, parents were worried that their child wouldn’t accept an organised activity that the parent suggested and they viewed organised activity as a way to limit the child’s excessive screen time. If the child participated in an activity, he or she could not use a screen during that time, without the parents having to enforce a limit for screen time.

#### Activity in everyday life is up to my child to engage in by him- or herself

Parents viewed activities in everyday life as being up to the child to engage in, where the child goes out to play with friends or siblings. Parents described how this was particularly frequent during the summer, but where the winter darkness or the child’s personal dislike for going out to be active could act as barriers. Here, parents also held the view that children are active enough during school time, which makes more activity unnecessary, especially when it is cold outside;

*When it’s cold then they are at home. And there is not so much space to move, but maybe the school has the role here. To do activities for the children. More than at home. But when the weather is good then they move a lot. (Father 3)*

#### Joint activity where it is up to my child to be active

Parents could organise joint activities, but where only the child is active and where it is up to the child to be active. The parent could take the child to the park, the beach or to an indoor play centre where the child is free to play as he or she wishes but where the parent does not participate, as one parent described;

*Sometimes we go to a shopping center and they play in the indoor play centre there. We sit down and have a coffee and they play. It costs a little but... (Father 5)*

#### Screen time according to my child’s decision and preference

Parents thought that it was the child who decided about screen time frequency or length, not them. A father expressed:

*He wants to watch tv and computer for such a long time, I want it to be less time, but I can’t say no. He gets addicted. One hour would feel good, then he can learn to read and write, I think that’s better. I want him to be ok with one hour [of screen]. (Father 6)*

Parents described how children either refused to obey limits, were aggressive towards the parents’ attempts to limit screen time or continued to watch a screen by stealth. Parents felt powerless and could not manage to keep limits despite attempting to, due to the strong will of the children, the parent’s lack of energy, or the parent’s many chores. Parents were worried about the consequences of screen time and how the child might be completely caught up in the screen, but also believed in being permissive and not enforcing prohibitions. They did not see why screen time should be limited if the child liked watching and if the child was active enough according to the parent.

## Discussion

This study explored variations in parents’ perceptions of their roles and responsibilities in relation to their child’s physical activity and sedentary behaviours in a Northern European context. The findings showed a variation of parental perceptions as a structural relationship involving different views of their own role for their child’s physical activity with three categories; “The parent decides”, “Parent-child interaction” and “The child/someone else decides”. Additionally, each category has four sub-categories that relate to practical parental approaches in different forms of specific activities; organised activity, activities in everyday life, joint activity and screen time. The results of this study further the understanding of parenting in relation to children’s physical activity and sedentary behaviours which is expected to have implications for the development of interventions to promote children’s physical activity.

This is the first study exploring this research topic in a Northern European context. A few previous studies have explored parents’ perceptions of their provision of healthy habits in general, including both mental and physical health for their children, in this context. In these studies, parents expressed a general view of having a high level of responsibility for their child’s development of healthy habits, e.g. through role modelling [55, 56]. The present study furthers the knowledge from these previous, broader studies by describing variation in parental views of their roles and responsibilities regarding children’s physical activity specifically.

### Findings in relation to parenting styles

The category “The parent decides” can be related to the authoritarian parenting style – high demandingness and low responsiveness – where the parent tells the child what to do, but does not show or teach them how to do it [28]. The category “child/someone else decides” can be related to the permissive parenting style – low demandingness and high responsiveness – where the child is allowed to act and behave as the child desires, with little interference or attempts to control from the parent [28]. The category “Parent-child interaction” can be related to the authoritative parenting style – high in both demandingness and responsiveness – as this category reflected parental behaviours which seemed to be highly responsive, yet with limits and demands. By letting the child explore their own boundaries, this category allowed the child to practice responsibility and independence, traits that are associated with this parenting style. Research has linked the authoritative parenting style to healthy child behaviours [22, 23, 31, 32], whereas the other styles have been related to unhealthy child behahviours [32, 33, 35]. Therefore, parents behaving in line with the categories “the parent decides” and “the child/someone else decides” could benefit from being supported by clinicians to move more towards guiding their children within a warm emotional atmosphere in accordance with the authoritative parenting style. Clinicians can listen for parental expressions in line with the two aforementioned categories and support parents in developing positive parenting practices, in line with the current evidence suggesting the importance of positive parenting for children’s healthy activity and sedentary behaviours [24, 25, 36, 37]. This could include motivating parents to adopt role modelling, or encourage physical activity, or set limits and rules for screen behaviour in a caring/respectful manner.

### Parental approaches in relation to organised activities

The results showed a variation in parental approaches to different forms of organised activities. Parents in the “parent-child interaction” category took their child’s personality, interests and preferences into account. Parents in the “parent decides” category were generally positive to organised activities, but the activities needed to suit the parent’s own preferences and perceptions. Among parents in the “child/someone else decides” category, organised activities were both described as unnecessary or as a good solution, if someone else other than the parent was responsible for activating the child. This has been found previously, where parents considered their children’s physical activity as primarily being the responsibility of the school [41]. Also, socioeconomic differences in how parents express responsibility has been shown in a qualitative study in New Zealand where parents with a low socioeconomic position tended to be reluctant towards encouraging their children to participate in organised activity that would require logistical or financial support from them [40].

Organised activities play an important role in promoting physical activity habits, physical and psychosocial health among children [57]. Previous research has shown a positive correlation between participation in organised physical activity and level of physical activity, where children that participate in organised activities are more likely to meet physical activity recommendations [57–59] and children are less likely to meet guidelines for physical activity when parents report more than four barriers to organised physical activity [60]. The present results are in line with previous research on common barriers and facilitators to organised physical activity such as lack of time, resources and accessibility [45], and socioeconomic inequalities with regards to participation in organised physical activity [9, 11].

In order to overcome inequalities in organised physical activity participation, it can be very important to take into account differences in parental perceptions of their own roles and barriers as well as cultural variations so that more people feel welcome to participate. It is important to acknowledge the differences in how parents perceive their role in their child’s participation in organised activity as presented in the current study, specifically considering that this study is conducted among parents in disadvantaged areas. Clinicians can support parents by motivating them according to how parents perceive their roles. Regarding parents in the “child/someone else decides” category, a clinician can highlight the benefits of organised physical activity and motivate parents by pointing out how the child can participate in an activity without the parent’s involvement, e.g. by being helped by a relative, or cooperating with other parents with children enrolled in the same activity. Clinicians can in other words motivate parents to resolve as many barriers as possible in order to make organised activity more likely for the child.

### Parental approaches in relation to activity in everyday life

The results also suggest that parents have different approaches to activity in everyday life. Parents in the “parent-child interaction” category tried to enhance possibilities for the child to be physically active, while parents in the “parent decides” category identified several barriers to physical activity, and the “child/someone else decides” category mostly thought that everyday activities were up to the child to engage in by themselves. To be active in everyday life is important for a healthy lifestyle. Even though it might not be feasible to overcome all barriers to activities in everyday life, active travel to school has been shown to be positively correlated with physical activity [61] and could be an activity where clinicians can motivate parents to encourage their children to engage in active transport at least 1 day of the week. Other potential interventions which may help parents to support their children’s physical activity include creating natural public meeting places for children to play during the winter [62, 63] as well as providing indoor opportunities for physical activity during cold weather [64]. Moreover, another important objective for future interventions may be to increase parents’ physical literacy, defined as “the motivation, confidence, physical competence, knowledge and understanding to value and take responsibility for engagement in physical activities for life” [65]. Our results showed that many parents felt unsure of their own ability to physically activate the child, especially during the winter. This may be especially relevant for parents who grew up outside of Sweden, who do not have the experience of winter activities. In order to increase parents’ confidence and to help them to see more possibilities of activities that can be done during winter, support from clinicians can be helpful.

### Parental approaches in relation to joint activities

The current study also identified that joint activities were perceived as something that was important, but often difficult to carry through. Parents in the “parent decides” category arranged activities according to their own preferences, and the “child/someone else decides” category arranged activities where the parents were not themselves active. The “parent-child interaction” category, on the other hand, emphasized interaction with the child. However, considering the well-known importance of parental support, role modelling and co-participation for physical activity habits among young children [66, 67], there is a need to inform parents about the importance of their own role for their child’s physical activity, and this is somewhere clinicians could support parental motivation. Thus, it may be useful to focus on the importance of being good role models, for example to participate together instead of being passive. It may also be feasible to provide parents with ideas on how to improve parent-child interaction. This study also elucidated differences in parental views regarding responsibility for physical activity. While the “parent-child interaction” category has a high sense of parental responsibility, parents in the category “child/someone else decides” view the child’s physical activity as the responsibility of someone else or the child. However, Wiltshire and Stevinson [68] have emphasized the need to further the knowledge in behavioural research by reconciling an individual perspective of responsibility and agency with a broader perspective of social structure. From this perspective, health inequalities, including socioeconomic differences in physical activity practices, can be understood in relation to class, culture and embodied dispositions towards different health practices.

### Parental approaches in relation to screen habits

In terms of screen habits, the results show that parents in the category “child/someone else decides”, expressed a lot of worry and frustration about their child’s screen habits, and felt powerless to find a solution and to establish and keep screen limits. Similarly, parents in the category “parent decides” felt lost even though they did set up limits for screen time. Parents in the category “parent-child interaction” managed to agree upon screen limit together with the child to a greater extent. A common issue among the parents was a need for more knowledge on how to relate to children’s screen time. Even though WHO recommends < 1 h screen time for children 3–4 years, there is a lack of clear guidelines on screen time for older children [69]. Specifically, there is a need for targeted information to different groups, including parents [70]. Current research differentiates between screen time for leisure and for education. However, the results in this study about screen time mostly related to leisure-based screen time use, although parents also mentioned the potential benefits of educational screen time. Previous research has also highlighted the importance of role modelling in terms of screen time habits [25], and in setting limits for screen use. Considering the results in the current study, it would be helpful for parents to get information, and motivational support, on how to relate to their child’s screen time habits, including how to be good role models, set limits and replace screen-based sedentary time with physically active time.

### Strength and limits of the study

A strength of the study was the approach used to analyse MI sessions. This material provided rich data where the participants reflected upon their lived experiences of the phenomenon of interest. In this study, previously recorded and transcribed MI sessions from the HSS intervention were used as unobtrusive, archival data, and the research question was posed afterwards. Unobtrusive data or semi-unobtrusive data do not influence the participant, and is a way of studying behaviours without the risk of reporting bias, which is often a potential problem when using interviews or questionnaires [50]. Having an MI session with a MI counsellor rather than an interview with a researcher may have created an environment where the parents could explore their own ideas, rather than trying to answer and reflect upon which approaches they use. Moreover, there can be many advantages of using already existing primary data, as re-analysis of data allows for the data to be explored more in-depth. On the other hand, the data from the MI sessions also included data that was unnecessary and irrelevant for the purposes of the present study.

Another strength of the study was the process of analysis. Having multiple people contributing to the analysis is a way to improve dependability and to reach a more comprehensive understanding of the data [71]. Furthermore, an audit trail with each step of the analysis was created to establish confirmability [71].

Quotes were used to illustrate the voice of the parents, although similar experiences were described by other parents. Phenomenography does not aspire to elucidate individual perceptions, but rather the variations and structural relationships within a group of people [54]. The categories and sub-categories describe the variations and structural relationship and the analysis was perceived to have reached saturation. The concept of transferability is used within qualitative research to ascertain whether the findings of a study can also be applied to other contexts outside of the study setting [71]. The findings of this study may be transferable to parents living in other areas of low socioeconomic position in high-income countries. However, evaluating transferability must be done on a case-to-case basis and is ultimately a judgment that must be done by the reader.

A limitation which may have narrowed the breadth of parental approaches represented is sampling. All the parents in our study identified physical activity as a problem area they would like to address in the MI session and parents that did not bring this up were excluded. This may have limited the scope of the study, as it would have been interesting to understand parental approaches to physical activity among those that did not choose to discuss it. It is possible that these parents could have fewer problems in this area, or that they do not see physical activity as equally important to discuss as diet for example. Moreover, the fact that the parents were participating in a health intervention may have influenced the selection of parents. As participation in the intervention was voluntary, including the MI session, parents who chose to participate may have already had an underlying motivation or interest towards improving health behaviours. Meanwhile, parents who declined to participate may have represented different attitudes and experiences, which were not captured in this study. However, the equal participation of both fathers and mothers in this is considered a strength of the study, as few previous studies in the area include fathers.

## Conclusions

This study contributes to a broader perspective on parental responsibility to support children’s physical activity. The results indicate a variation in how parents perceive their roles and responsibilities for their children’s physical activity and sedentary behaviour related to specific types of activities. Furthermore, the study indicates areas where parents need support regarding how to guide their child and how parental responsibility can have a positive influence on children’s physical activity and sedentary behaviours. This knowledge is important to further understanding on how to support parents in terms of advice and practical examples on how to guide and take a greater responsibility for their children’s physical activity and screen time habits. Furthermore, the study is expected to facilitate health-promoting initiatives in disadvantaged areas.

## Data Availability

The transcripts generated and analysed during the current study are not available in order to maintain participant confidentiality. Further information, such as documentation of the analysis, are available from the corresponding author on reasonable request.

## Abbreviations

HSS: A Healthy School Start
MI: Motivational interviewing
WHO: World Health Organization

## References

[1] Janssen I, Leblanc AG (2010). Systematic review of the health benefits of physical activity and fitness in school-aged children and youth. Int J Behav Nutr Phys Act.

[2] Wu XY, Han LH, Zhang JH (2017). The influence of physical activity, sedentary behavior on health-related quality of life among the general population of children and adolescents: A systematic review. PLoS One.

[3] Spruit A, Assink M, van Vugt E (2016). The effects of physical activity interventions on psychosocial outcomes in adolescents: A meta-analytic review. Clin Psychol Rev.

[4] Lubans D, Richards J, Hillman C, et al. Physical Activity for Cognitive and Mental Health in Youth: A Systematic Review of Mechanisms. Pediatrics. 2016;138. 2016/08/21. 10.1542/peds.2016-1642. 10.1542/peds.2016-1642 27542849

[5] Warburton DER, Bredin SSD (2017). Health benefits of physical activity: a systematic review of current systematic reviews. Curr Opin Cardiol.

[6] Liu M, Wu L, Ming Q (2015). How Does Physical Activity Intervention Improve Self-Esteem and Self-Concept in Children and Adolescents? Evidence from a Meta-Analysis. PLoS One.

[7] Ekeland E, Heian F, Hagen KB (2005). Can exercise improve self esteem in children and young people? A systematic review of randomised controlled trials. Br J Sports Med.

[8] WHO (2020). WHO guidelines on Physical activity and sedentary behaviour.

[9] Nyberg G. Få unga rör sig tillräckligt. In: Centrum för idrottsforskning, editor. *De aktiva och de inaktiva: Om ungas rörelse i skola och på fritid*. Stockholm: TMG Tabergs; 2017.

[10] Guthold R, Stevens GA, Riley LM (2018). Worldwide trends in insufficient physical activity from 2001 to 2016: a pooled analysis of 358 population-based surveys with 1.9 million participants. Lancet Global Health.

[11] Love R, Adams J, Atkin A (2019). Socioeconomic and ethnic differences in children's vigorous intensity physical activity: a cross-sectional analysis of the UK Millennium Cohort Study. BMJ open.

[12] Wijtzes AI, Jansen W, Bouthoorn SH (2014). Social inequalities in young children’s sports participation and outdoor play. Int J Behav Nutr Phys Act.

[13] Blomdahl UES, Bergmark K, Lengheden L, Åkesson M (2019). Ökar ojämlikheten i föreningsidrotten? – en studie om socioekonomisk bakgrund och barns och ungdomars deltagande i idrottsförening.

[14] Beckvid Henriksson G, Franzén S, Elinder LS, et al. Low socio-economic status associated with unhealthy weight in six-year-old Swedish children despite higher levels of physical activity. Acta Paediatr. 2016;105:1204–1210. 2016/03/24. 10.1111/apa.13412. 10.1111/apa.13412 27008097

[15] Biddle SJH, Atkin AJ, Cavill N, Foster C (2011). Correlates of physical activity in youth: a review of quantitative systematic reviews. Int Rev Sport Exerc Psychol.

[16] Sallis JF, Prochaska JJ, Taylor WC (2000). A review of correlates of physical activity of children and adolescents. Med Sci Sports Exerc.

[17] Telama R (2009). Tracking of physical activity from childhood to adulthood: a review. Obesity facts.

[18] Trost SG, Loprinzi PD (2011). Parental influences on Physical activity behavior in children and adolescents: a brief review. Am J Lifestyle Med.

[19] Utter J, Denny S, Robinson E (2011). Social and physical contexts of schools and neighborhoods: associations with physical activity among young people in New Zealand. Am J Public Health.

[20] Davison KK, Nishi A, Kranz S (2012). Associations among social capital, parenting for active lifestyles, and youth physical activity in rural families living in upstate New York. Soc Sci Med.

[21] Rhodes RE, Guerrero MD, Vanderloo LM (2020). Development of a consensus statement on the role of the family in the physical activity, sedentary, and sleep behaviours of children and youth. Int J Behav Nutr Phys Act.

[22] Sleddens EF, Gerards SM, Thijs C (2011). General parenting, childhood overweight and obesity-inducing behaviors: a review. Int J Pediatr Obes.

[23] Yaffe Y (2018). Physical Activity Among Israeli-Arab Adolescent Males: How Do Parenting Styles Matter?. Am J Men’s Health.

[24] Hutchens A, Lee RE (2018). Parenting Practices and Children’s Physical Activity: An Integrative Review. J Sch Nurs.

[25] Xu H, Wen LM, Rissel C (2015). Associations of parental influences with physical activity and screen time among young children: a systematic review. J Obes.

[26] Heitzler CD, Martin SL, Duke J, Huhman M (2006). Correlates of physical activity in a national sample of children aged 9–13 years. Prev Med.

[27] Thompson JL, Jago R, Brockman R (2010). Physically active families - de-bunking the myth? A qualitative study of family participation in physical activity. Child Care Health Dev.

[28] Baumrind D (1966). Effects of authoritative parental control on child behavior. Child Dev.

[29] Masse LC, O'Connor TM, Tu AW (2017). Conceptualizing physical activity parenting practices using expert informed concept mapping analysis. BMC Public Health.

[30] Darling N, Steinberg L (1993). Parenting style as context: an integrative model. Psychol Bull.

[31] Draper CE, Grobler L, Micklesfield LK (2015). Impact of social norms and social support on diet, physical activity and sedentary behaviour of adolescents: a scoping review. Child Care Health Dev.

[32] Van der Geest KE, Mérelle SYM, Rodenburg G (2017). Cross-sectional associations between maternal parenting styles, physical activity and screen sedentary time in children. BMC Public Health.

[33] Hennessy E, Hughes SO, Goldberg JP (2010). Parent-child interactions and objectively measured child physical activity: a cross-sectional study. Int J Behav Nutr Phys Act.

[34] Schary DP, Cardinal BJ, Loprinzi PD (2012). Parenting style associated with sedentary behaviour in preschool children. Early Child Dev Care.

[35] Langer SL, Crain AL, Senso MM (2014). Predicting child physical activity and screen time: parental support for physical activity and general parenting styles. J Pediatr Psychol.

[36] Sleddens EFC, Gubbels JS, Kremers SPJ, van der Plas E, Thijs C (2017). Bidirectional associations between activity-related parenting practices, and child physical activity, sedentary screen-based behavior and body mass index: a longitudinal analysis. Int J Behav Nutr Phys Act.

[37] De Lepeleere S, De Bourdeaudhuij I, Van Stappen V (2018). Parenting practices as a mediator in the association between family socio-economic status and screen-time in primary schoolchildren: a Feel4Diabetes study. Int J Environ Res Public Health.

[38] Best K, Ball K, Zarnowiecki D, et al. In Search of Consistent Predictors of Children’s Physical Activity. Int J Environ Res Public Health. 2017;14. 2017/10/21. 10.3390/ijerph14101258. 10.3390/ijerph14101258 PMC5664759 29053612

[39] Brockman R, Jago R, Fox KR (2009). “Get off the sofa and go and play”: family and socioeconomic influences on the physical activity of 10–11 year old children. BMC Public Health.

[40] Cox M, Schofield G, Kolt GS (2010). Responsibility for children’s physical activity: Parental, child, and teacher perspectives. J Sci Med Sport.

[41] Trigwell J, Murphy RC, Cable NT, Stratton G, Watson PM (2015). Parental views of children’s physical activity: a qualitative study with parents from multi-ethnic backgrounds living in England. BMC Public Health.

[42] Mâsse LC, O'Connor TM, Tu AW (2016). Are the Physical activity parenting practices reported by US and Canadian parents captured in currently published instruments?. J Phys Act Health.

[43] Bergmeier H, Skouteris H, Hetherington M (2015). Systematic research review of observational approaches used to evaluate mother-child mealtime interactions during preschool years. Am J Clin Nutr.

[44] Marton FB, S. (1997). Learning and awareness.

[45] Hesketh KR, Lakshman R, van Sluijs EMF (2017). Barriers and facilitators to young children’s physical activity and sedentary behaviour: a systematic review and synthesis of qualitative literature. Obes Rev.

[46] Roubinov DS, Boyce WT (2017). Parenting and SES: relative values or enduring principles?. Curr Opin Psychol.

[47] Nyberg G, Norman A, Sundblom E, Zeebari Z, Elinder LS (2016). Effectiveness of a universal parental support programme to promote health behaviours and prevent overweight and obesity in 6-year-old children in disadvantaged areas, the healthy school start study II, a cluster-randomised controlled trial. Int J Behav Nutr Phys Act.

[48] Ministry of Employment Sweden. Urban Development Areas (In Swedish: Urbana utvecklingsområden. Statistisk uppföljning utifrån sju indikatorer). Stockholm: Goverment Offices of Sweden; 2012.

[49] Nyberg G, Sundblom E, Norman A, Elinder LS (2011). A healthy school start - parental support to promote healthy dietary habits and physical activity in children: design and evaluation of a cluster-randomised intervention. BMC Public Health.

[50] Webb EJ, Campbell DT, Schwartz RD, Sechrest L (2000). Unobtrusive measures.

[51] Galobardes B, Shaw M, Lawlor DA (2006). Indicators of socioeconomic position (part 1). J Epidemiol Commun Health (1979).

[52] Miller WR, Rollnick S (2013). Motivational interviewing: Helping people change.

[53] Åkerlind GS, Bowden J, Green P (2005). Phenomenographic methods: a case illustration. Doing developmental phenomenography.

[54] Åkerlind GS (2012). Variation and commonality in phenomenographic research methods. High Educ Res Dev.

[55] Gunnarsdottir H, Povlsen L, Ringsberg KC (2017). Health lifestyles of pre-school children in Nordic countries: parents’ perspectives. Health Promot Int.

[56] Stenhammar C, Wells M, Ahman A (2012). ‘Children are exposed to temptation all the time’- parents’ lifestyle-related discussions in focus groups. Acta Paediatr.

[57] Eime RM, Young JA, Harvey JT (2013). A systematic review of the psychological and social benefits of participation in sport for adults: informing development of a conceptual model of health through sport. Int J Behav Nutr Phys Act.

[58] Josef M, Petra Nováková L, Krzysztof S (2012). The association between participation in organised physical activity and level of physical activity and inactivity in adolescent girls [Vztah mezi organizovanou pohybovou aktivitou a úrovní pohybové aktivity a inaktivity u adolescentních dívek]. Acta Universitatis Palackianae Olomucensis Gymnica.

[59] Marques A, Ekelund U, Sardinha LB (2015). Associations between organized sports participation and objectively measured physical activity, sedentary time and weight status in youth. J Sci Med Sport.

[60] Smith BJ, Grunseit A, Hardy LL (2010). Parental influences on child physical activity and screen viewing time: a population based study. BMC Public Health.

[61] Schoeppe S, Duncan MJ, Badland H (2013). Associations of children’s independent mobility and active travel with physical activity, sedentary behaviour and weight status: a systematic review. J Sci Med Sport.

[62] Smith M, Hosking J, Woodward A (2017). Systematic literature review of built environment effects on physical activity and active transport - an update and new findings on health equity. Int J Behav Nutr Phys Act.

[63] Katapally TR, Rainham D, Muhajarine N (2016). The Influence of Weather Variation, Urban Design and Built Environment on Objectively Measured Sedentary Behaviour in Children. AIMS Public Health.

[64] Tucker P, Gilliland J (2007). The effect of season and weather on physical activity: a systematic review. Public Health.

[65] Whitehead M. Definition of physical literacy and clarification of related issues. ICSSPE Bull. 2013;65:29–34.

[66] Gustafson SL, Rhodes RE (2006). Parental correlates of physical activity in children and early adolescents. Sports Med.

[67] Tucker P, van Zandvoort MM, Burke SM (2011). The influence of parents and the home environment on preschoolers’ physical activity behaviours: a qualitative investigation of childcare providers’ perspectives. BMC Public Health.

[68] Wiltshire G, Stevinson C (2018). Exploring the role of social capital in community-based physical activity: qualitative insights from parkrun. Qual Res Sport Exer Health.

[69] World Health Organisation (2019). Guidelines on physical activity, sedentary behaviour and sleep for children under 5 years of age.

[70] Saunders TJ, Vallance JK (2017). Screen Time and Health Indicators Among Children and Youth: Current Evidence, Limitations and Future Directions. Appl Health Econ Health Policy.

[71] Patton M (2015). Qualitative research and evaluation methods.

